# Revised minimal important difference values for the visual analogue scale and Foot Health Status Questionnaire when used for plantar heel pain

**DOI:** 10.1002/jfa2.70021

**Published:** 2024-12-16

**Authors:** Karl B. Landorf, Georgia N. Twyford, Matthew P. Cotchett, Glen A. Whittaker

**Affiliations:** ^1^ Discipline of Podiatry School of Allied Health, Human Services and Sport La Trobe University Bundoora Victoria Australia; ^2^ Warrnambool Podiatry Group Warrnambool Victoria Australia

**Keywords:** Foot Health Status Questionnaire, minimal important difference, patient reported outcome measures, plantar fasciitis, plantar heel pain, visual analogue scale

## Abstract

**Background:**

The visual analogue scale (VAS) and the Foot Health Status Questionnaire (FHSQ) are patient‐reported outcome measures that are frequently used to evaluate the management of plantar heel pain. This study aimed to re‐calculate (i.e. revise) the minimal important differences (MIDs) of the VAS and the FHSQ when used for plantar heel pain to enhance the validity and precision of previous estimates.

**Methods:**

This study used an anchor‐based method to calculate MIDs and incorporated best‐practice analyses to ensure credibility of the estimates. Data from 369 participants previously recruited from the community into four randomised controlled trials that evaluated interventions for plantar heel pain were used. VAS and FHSQ data from these participants at baseline and follow‐up were pooled to calculate the MIDs (95% confidence intervals). A 15‐point global rating of change Likert scale was used at follow‐up as the transition scale, which was anchored to baseline. For the VAS, MIDs for two distinct types of pain were calculated: average pain and first‐step pain. For the FHSQ, MIDs for two domains were calculated: foot pain and foot function.

**Results:**

The revised MIDs for the 100 mm VAS were −8.5 mm (95% CI: −12.2 to −4.7) for average pain and −19.2 mm (95% CI: −24.7 to −13.8) for first‐step pain, which represent improvements in pain. The MIDs for the FHSQ were 12.4 points (95% CI: 6.9 to 18.0) for foot pain and 6.4 points (95% CI: 0.9 to 11.9) for foot function, which represent improvements in foot health status.

**Conclusions:**

The revised MIDs from this study for the VAS and the FHSQ when used for plantar heel pain have enhanced validity and precision compared to previous estimates. This is important for clinicians and researchers as it provides a better understanding of how much improvement is required by an individual before an important change is experienced. The revised MIDs will also assist researchers with prospective sample size calculations, so future clinical trials are appropriately powered from a statistical standpoint.

## 1 INTRODUCTION

Plantar heel pain, also referred to as plantar fasciitis or plantar fasciopathy, is a condition characterised by localised pain on the underside of the heel [1, 2]. Although population prevalence figures vary depending on the population studied, prevalence has been estimated to be up to 10% [3, 4, 5, 6, 7, 8, 9]. Further, one study from the United Kingdom found that the prevalence of plantar heel pain *classified as disabling* was nearly 8% [7]. Until recently, the clinical course of this condition was thought to be self‐limiting over approximately 12 months [1], but a cohort study published in 2018 from Denmark found that symptoms can persist for many years, with 45% of the cohort still experiencing symptoms 10 years after the initial onset of symptoms [10]. Plantar heel pain leads to a high economic burden [11, 12, 13] and significantly poorer health‐related quality of life [14, 15].

Treatment of plantar heel pain is frequently multifaceted, with no one intervention being found to be the most beneficial [16, 17, 18, 19]. When interventions are implemented for plantar heel pain, establishing whether they provide important benefits for patients is fundamental, not only clinically but also from a statistical standpoint in research [20, 21, 22]. The importance of an intervention can be determined by using patient‐reported outcome measures both in clinical practice as well as in clinical trials such as randomised controlled trials (RCTs). When considering the findings of patient‐reported outcome measures, the minimal important difference (MID) is a key indicator of whether an intervention provides an important effect on a patient's health status (i.e., whether a patient perceives the intervention has improved their health). Therefore, the MID represents the smallest change in health status that is regarded as important by the patient [22]. Not only does this critical indicator relate to generic patient‐reported outcome measures (i.e. measures that assess general health status) but it also relates to disease‐ or region‐specific outcome measures such as those used for plantar heel pain.

Two previous studies by the first author, which were published in 2008 [23] and 2010 [24], attempted to estimate MIDs for two patient reported outcome measures that are frequently used for plantar heel pain—the 100 mm visual analogue scale (VAS) and the Foot Health Status Questionnaire (FHSQ). The VAS is often used to measure pain [25], whereas the FHSQ is a more complex instrument that measures foot health status [26]. The 2010 study [24] was an improvement on the 2008 study [23] as it used better methods; however, it still had a limited sample size, which led to relatively wide confidence intervals (CIs). As a consequence, this means that the MID estimates had less than optimal precision; put simply, there was more uncertainty in the estimates than we wanted. In addition, both studies did not use best‐practice analyses to optimise the credibility of the MID estimates [27]. Accordingly, additional research using best‐practice analyses and a larger sample size is likely to enhance both the validity and precision of these previous estimates. Doing so would allow greater confidence in what patients perceive as an important change in clinical practice and provide researchers with more precise data for pre‐specifying sample sizes in clinical trials.

Therefore, this study aimed to re‐calculate (i.e. revise) the MIDs of the VAS and the FHSQ when used for plantar heel pain to enhance the validity and precision of previous estimates.

## 2 METHODS

### 2.1 Study design

This study was explicitly designed to calculate MIDs, so it used methods that were optimal for that task namely, an ‘anchor‐based method’ [28, 29]. This study incorporated best‐practice data analysis to ensure credibility of the estimates [27]. Data for this study were taken from four RCTs that evaluated interventions for plantar heel pain [30, 31, 32, 33]. All RCTs used similar methods—the same general protocol and outcome measures—which have been reported in detail elsewhere [30, 31, 32, 33]. Relevant data from these RCTs were extracted for this study to determine the MIDs for the VAS and the FHSQ.

### 2.2 Ethical approval

Ethical approval was obtained from the relevant institutional ethics committees (University of Western Sydney Ethics Review Committee (Human Subjects)—approval # HREC 04/157 [30, 31], La Trobe University Research Ethics Committee—approval # 09–062 [32], La Trobe University Human Ethics Committee—approval # 15–120 [33]), and all participants gave written informed consent to participate in the original RCTs.

### 2.3 Participants

Between 2006 and 2019, 369 participants with plantar heel pain were recruited from the general public to take part in the four RCTs, which evaluated the effectiveness of four non‐surgical interventions for plantar heel pain: (i) low‐Dye taping [30], (ii) calf muscle stretching [31], (iii) corticosteroid injection [32], and (iv) prefabricated foot orthoses [33]. In all the RCTs, participants had a clinical diagnosis of plantar heel pain, which they needed to have experienced for at least 4 weeks. In one of the RCTs [32], which evaluated corticosteroid injection, an additional eligibility criterion was participants were required to have a plantar fascia thickness of 4.0 mm or greater when measured on an ultrasound scan. To avoid confounding of plantar heel pain that could have been associated with other conditions or diseases, individuals were excluded from all RCTs if they had inflammatory, osseous, metabolic or neurological disorders. This exclusion criterion was applied so that participants were most likely to have isolated plantar heel pain (e.g. of mechanical origin) and not plantar heel pain caused by a secondary disorder that had widespread effects on the body (e.g. inflammatory arthritis), which may not have benefitted from the interventions that were evaluated in the RCTs. They were also excluded if they had received a corticosteroid injection within the past 3 months. Additional specific inclusion and exclusion criteria for each RCT can be viewed in each publication [30, 31, 32, 33]. A summary of participant characteristics—which have already been published in detail [30, 31, 32, 33]—is included in Table 1.

**TABLE 1:** Participant characteristics of the four RCTs that data were accessed to calculate MIDs.

RCT authors, date	Interventions	Sample size	Age in years Mean (SD), range	Sex *n* (%) women	BMI in kg/m^2^ Mean (SD), range	Duration of symptoms in months^a^ Median (IQR)
Radford et al, 2006 [30]	Low‐Dye taping versus placebo	92	50 (14)^b^	55 (60)	30 (6)^b^	10 (2–240)^c^
Radford et al, 2007 [31]	Calf muscle stretching versus placebo	92	50 (11), 24–80	56 (61)	32 (6), 20–48	13 (7–25)^c^
McMillan et al, 2012 [32]	Corticosteroid injection versus placebo	82	53 (11), 25–74	39 (48)	31 (5), 22–48	9 (5–14)
Whittaker et al, 2019 [33]	Prefabricated FOs versus corticosteroid injection	103^d^	44 (12), 21–72	63 (61)	30 (6), 17–48	6 (4–12)

Abbreviations: BMI, body mass index; FOs, foot orthoses; IQR, interquartile range; MID, minimal important difference; RCT, randomised controlled trial; SD, standard deviation.

^a^ Duration of symptoms in all RCTs was skewed, so median and interquartile range (IQR) are reported.

^b^ Range was not reported and could not be calculated.

^c^ RCT reported range (not IQR) for duration of symptoms.

^d^ RCT initially enrolled 103 participants but 7 of these did not complete the 15‐point global rating of change Likert scale (the ‘anchor’) at their four‐week appointment, so they could not be included in this study (i.e. MIDs could not be calculated for these participants).

### 2.4 Outcome measures

The outcome measures of interest were evaluated in the four RCTs, and subsequently, these data were used in this study. The outcome measures of interest were the 100 mm VAS and the FHSQ, which are used to measure pain levels [34, 35, 36, 37] and foot health status [26], respectively. The outcomes were measured at the baseline and follow‐up appointments. Regarding the follow‐up appointments, the outcomes were assessed at different times within each RCT, but all measurements used in this study represent *short‐term* assessments after 1 week [30], 2 weeks [31] and 4 weeks [32, 33]. Overall, these follow‐up assessments reflect a suitably short timeframe for participants to remember their health status at baseline (i.e. this timeframe is suitably short to avoid recall issues about their health status prior to intervention) [27].

In relation to the VAS, two distinct types of pain were assessed: 'average pain' (over the last 7 days) and 'first‐step pain' (a typical complaint with plantar heel pain, manifesting as pain when taking the first few steps in the morning or after a periods of inactivity [1, 38]). In relation to the FHSQ, foot health status was assessed for four domains: 'foot pain', 'foot function', 'footwear' and 'general foot health (GFH)' [26].

For the VAS, a 100 mm horizontal scale was used where *lower* scores indicate less pain [25]. In this study, the terminal descriptors ‘no pain’ and ‘worst pain imaginable’ were used on the far left (i.e. 0 mm) and far right (i.e. 100 mm) of the scale, respectively. For the FHSQ, a 100 point scale is used but *higher* scores indicate better foot health (i.e. 0 = worst foot and 100 = best foot health). Therefore, for the VAS, the ‘best’ score is 0, whereas for the FHSQ, the ‘best’ score is 100 that is (i.e. the FHSQ is reverse‐scored compared to the VAS)

### 2.5 Calculation of the MIDs

We set out to calculate the MIDs for two types of pain measured with the VAS (average pain and first‐step pain) and for all four domains of the FHSQ (foot pain, foot function, footwear and GFH). An anchor‐based method was used to calculate the MIDs—this method has been reported to be the most appropriate to determine MIDs [27] and is considered a better approach than distribution‐based methods [28, 29].

**FIGURE 1:**
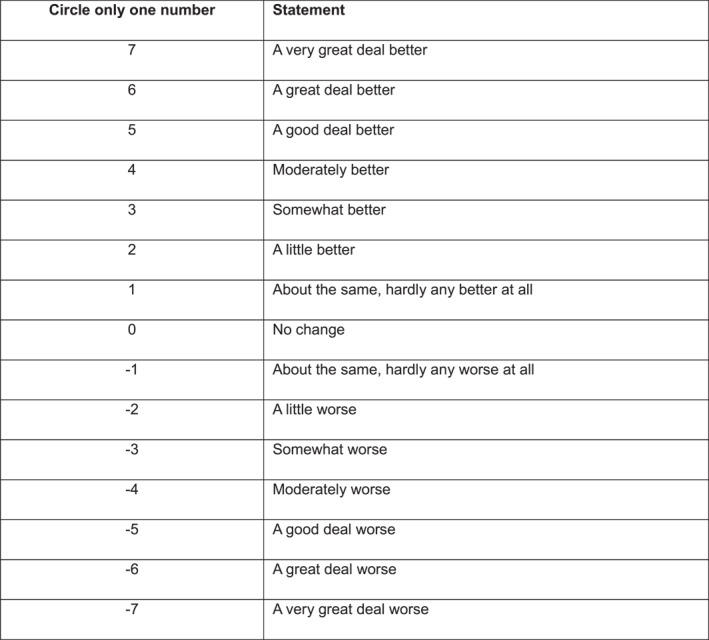
The 15‐point global rating of change Likert scale used in each randomised controlled trial.

To calculate the MIDs using an anchor‐based approach, a global rating of change scale was used, which is also referred to as a transition rating scale [27]. In our study, we used a 15‐point global rating of change Likert scale (Figure 1). Participants completed this scale at their follow‐up appointment by comparing their pain or health status to baseline (thus providing an ‘anchor’ for the scale [39]), which was completed independent of the VAS and FHSQ. This scale states levels of change in simple terms from a study participant's (or clinically from a patient's) perspective. Each number (or point) on the 15‐point scale represents a different change in health status that is readily interpretable by participants (i.e. understandable and relevant) [27]. The numbers on the scale range from −7 to +7. The value −7 represents a change in health status that is considered by a participant as 'a very great deal worse', whereas +7 indicates a change that is 'a very great deal better'. The value of 0 represents 'no change' in health status. For this study, participants who answered '+2 and +3' represented a 'small change' (or minimal change) in health status and participants who answered '0 and +1' represented 'no change' in health status [24]. The ‘no change’ group needs to also be taken into account to correct for the change in outcome scores in this group [40].

To ensure credibility, Devji and colleagues [27] recommend that prior to an MID calculation, the correlation is considered between the anchor (in this study, the 15‐point global rating of change Likert scale) and each outcome variable the MID is planned to be calculated for. Therefore, the score recorded on the anchor should be appreciably correlated with the outcome measure score (in this study the VAS or FHSQ) at follow‐up and the change in the outcome measure score from an initial assessment to follow‐up, and ideally, these correlations should be approximately equal and opposite [27]. To achieve this, Spearman's rank correlation coefficients were calculated (we assumed that the 15‐point global rating of change Likert scale provides ordinal scale data and the VAS and FHSQ provides continuous scale data). Based on the recommendation by Devji and colleagues [27], we chose a moderate to high correlation coefficient of at least ±0.5 to be appreciably correlated that is (i.e. a correlation that could be considered acceptable). Prior to checking the correlations, 7 participants who had missing outcome measures or global rating of change scale data were removed from the analysis so that the sample size used for the correlation and MID analyses was 362.

**FIGURE 2:**
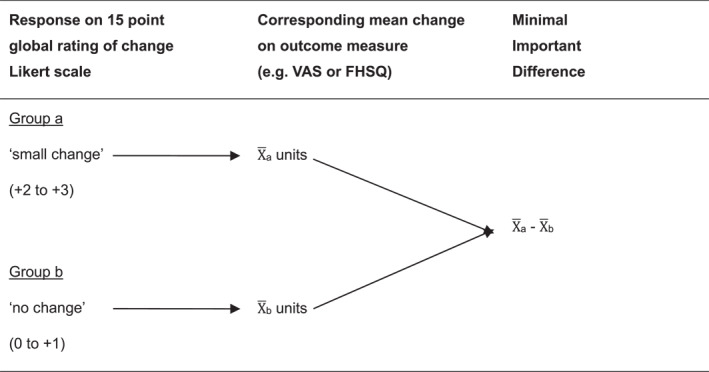
Method used to calculate the MID using the anchor‐based approach. FHSQ, Foot Health Status Questionnaire; MID, minimal important difference; VAS, visual analogue scale; $\bar{X}$_a_, mean for group a; $\bar{X}$_b_, mean for group b.

Following establishment that variables were appreciably correlated, we calculated the MID. To achieve this, we isolated all participants who reported a 'small change' and 'no change' on the 15‐point global rating of change Likert scale into two separate groups; Group a and Group b, respectively. Following this, the mean change in both outcome measures (VAS and FHSQ) from baseline was calculated for each group. To calculate the MID, the mean change in an outcome measure for the participants who reported 'no change' (Group b) was subtracted from the mean change in the same outcome measure for the participants who reported a 'small change' (Group a) as illustrated in Figure 2. This methodology was similar to that used previously by Landorf and colleagues [24].

Means, standard deviations (SDs) and mean change for each outcome measure were calculated using IBM SPSS Statistics version 29.0. To assess the precision of MID estimates, 95% CIs were calculated using the PEDro CI calculator [41]. As we calculated parametric statistics (e.g. mean), all outcome data were initially checked for normality to satisfy the necessary statistical assumptions. Variables demonstrating distributions that were not normal were then assessed for outliers (i.e. participants that had scores ±3 SDs from the mean) and those participants were removed from the analysis of that variable to ensure a normal distribution [42]. A full step‐by‐step guide for calculating MIDs for patient‐reported outcome measures is included in Supporting Information S1.

## 3 RESULTS

There was appreciable correlation between the 15‐point global rating of change scale and four of the outcome measures (VAS average and first‐step pain and the FHSQ pain and function domains). These appreciable correlations were found at follow‐up and for the change in the outcome measure score from initial assessment to follow‐up. Furthermore, the correlations were approximately equal and opposite. Two domains of the FHSQ (footwear and GFH) were not appreciably correlated with the 15‐point global rating of change Likert scale. Results of the correlation analyses are presented in Table 2.

**TABLE 2:** Results of the correlation analyses between the 15‐point global rating of change Likert scale and each outcome measure.

Outcome	Domain	Time	Spearman's rho (95% CI)	*n* ^a^	*p*‐value
VAS	Average pain	T1	0.142 (0.020 to 0.259)	276	0.019
		T2	−0.626 (−0.695 to −0.546)	276	<0.001
		∆	0.785 (0.733 to 0.827)	276	<0.001
	First‐step pain	T1	−0.041 (−0.146 to 0.066)	359	0.443
		T2	−0.680 (−0.734 to −0.619)	359	<0.001
		∆	0.724 (0.669 to 0.771)	359	<0.001
FHSQ	Pain	T1	0.137 (0.031 to 0.240)	362	0.009
		T2	0.748 (0.697 to 0.791)	362	<0.001
		∆	−0.676 (−0.730 to −0.614)	362	<0.001
	Function	T1	0.019 (−0.087 to 0.125)	362	0.722
		T2	0.502 (0.418 to 0.577)	362	<0.001
		∆	−0.554 (−0.623 to −0.476)	362	<0.001
	Footwear	T1	0.052 (−0.070 to 0.172)^b^	278	0.389
		T2	−0.097 (−0.216 to 0.024)^b^	278	0.106
		∆	0.200 (0.081 to 0.313)^b^	278	<0.001
	GFH	T1	0.117 (−0.003 to 0.235)^b^	280	0.050
		T2	0.398 (0.291 to 0.495)^b^	280	<0.001
		∆	−0.310 (−0.415 to −0.196)^b^	280	<0.001

*Note*: 1. Correlation coefficients of at least ±0.5 were considered to be appreciably correlated for the MID of an outcome measure to be subsequently calculated. 2. The global rating of change scale score should be appreciably correlated with the outcome measure score at follow‐up (i.e. T2) and the change in the outcome measure score from T1 to T2 (i.e. ∆), and ideally, these correlations should be approximately equal and opposite [27].

Abbreviations: CI, confidence interval; FHSQ, Foot Health Status Questionnaire; GFH, general foot health; T1, Baseline (i.e., prior to intervention); T2, follow‐up (i.e., after intervention); VAS, visual analogue scale; ∆, change in the outcome measure score from T1 to T2.

^a^ *n* for each domain varies as one of the RCTs that was included did not measure certain domains and some domains were not normally distributed, so outliers that were 3 SDs from the mean were removed.

^b^ indicates values that did not reach an appreciable correlation.

Once it was determined that the VAS average pain and first step pain and the FHSQ pain and function domains were appreciably correlated with the 15‐point global rating of change Likert scale, their MIDs were calculated. As the FHSQ footwear and GFH domains were not appreciably correlated with the 15‐point global rating of change Likert scale, we did not progress to calculate their MIDs. For the VAS, the MIDs were −8.5 mm (95% CI: −12.2 to −4.7) for average pain and −19.2 mm (95% CI: −24.7 to −13.8) for first‐step pain—these values represent a reduction in pain. For the FHSQ, the MIDs were 12.4 points (95% CI: 6.9 to 18.0) for foot pain and 6.4 points (95% CI: 0.9 to 11.9) for foot function—these values represent an improvement in foot health status. The key data associated with the MID calculations for the VAS and the FHSQ are presented in Table 3.

**TABLE 3:** Anchor‐based calculations of MIDs with 95% confidence interval (CI) for the VAS and the FHSQ.

Outcome	Domain	+2 to +3 group		0 to +1 group		MID values	95% CI
		Mean change (SD)	*n*	Mean change (SD)	*n*		
VAS	Average pain	−10.04 (11.49)	58	−1.56 (8.78)	59	−8.5	−12.2 to −4.7
	First‐step pain	−23.22 (22.59)	77	−3.98 (8.70)	77	−19.2	−24.7 to −13.8
FHSQ	Pain	20.795 (18.178)	77	8.356 (16.956)	81	12.4	6.9 to 18.0
	Function	11.769 (17.443)	77	5.401 (17.588)	81	6.4	0.9 to 11.9

*Note*: 1. For the VAS, a negative mean change value indicates a reduction in pain from baseline to follow‐up, and for the FHSQ, a positive mean change value indicates an improvement in foot health from baseline to follow‐up. 2. The *n* for each domain varies as one of the RCTs that data were taken from did not measure certain domains, and some domains were not normally distributed, so outliers that were 3 SDs from the mean were removed. 3. MID values represent the difference between the mean of participants who indicated +2 or +3 (small change) and the mean of participants who indicated 0 or +1 (no change) on the 15‐point global rating of change Likert scale. 4. MID and 95% CI values have been rounded down to one decimal point on purpose (i.e. to be appropriate for the level of measurement).

Abbreviations: CI, confidence interval; FHSQ, Foot Health Status Questionnaire; MID, minimal important difference; SD, standard deviation; VAS, visual analogue scale.

## 4 DISCUSSION

The VAS and the FHSQ are two patient‐reported outcome measures that are frequently used in foot‐related research, and the MIDs for these measures are a key indicator when determining if an intervention has provided an important effect for a patient. With respect to plantar heel pain, the MIDs for the VAS and the FHSQ have been estimated twice previously by the first author and these findings were published in 2008 [23] and 2010 [24]. The earlier study [23] was limited by its sampling methods, which may have led to a decreased variability in the sample and false precision in the MID estimates. The latter study [24], although it used better sampling methods, was limited by a sub‐optimal sample size as it pooled data from only two RCTs, which led to relatively wide CIs for the MID estimates. In addition, the two studies [23, 24] did not check whether the anchor (i.e. a 15‐point global rating of change Likert scale) and each outcome variable that the MID was calculated for were appreciably correlated, which is a best‐practice recommendation [27] that was published after these two studies. Therefore, the present study aimed to improve on these limitations to provide more valid and precise estimates of the MIDs. To achieve this, we used best‐practice methods and a larger sample size, which has resulted in enhanced validity and precision.

**FIGURE 3:**
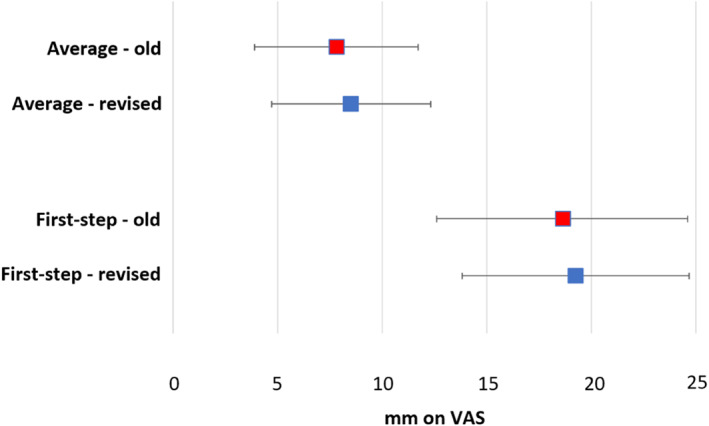
Comparisons of the old and revised MIDs for the VAS for average pain and first‐step pain. MID, minimal important difference; VAS, visual analogue scale.

For the 100 mm VAS, the MID was found to be −8.5 mm (i.e. a reduction in pain of 8.5 mm for a patient) for average pain and −19.2 mm (i.e., a reduction in pain of 19.2 mm) for first‐step pain. Average pain is generally considered the average pain for a patient over the last 7 days (or week) and is considered a more valuable measure than current pain [43]. First‐step pain is the pain experienced after a long period of non‐weightbearing (e.g. upon first stepping out of bed in the morning) and is a typical complaint of individuals with plantar heel pain [1, 38]. These findings indicate that on a 100 mm VAS, a reduction of 8.5 mm for average pain and 19.2 mm for first‐step pain is needed for a patient to recognise a minimal but important change in their pain level. These MID values are comparable to our previous study that found MID values of −7.8 mm for average pain and −18.6 mm for first‐step pain when used for people with plantar heel pain [24]. Although the differences between the estimates from this study compared with our previous study are small, the widths of the 95% CIs for these MIDs have reduced from 7.8 to 7.5 (a reduction of 0.3 mm) for average pain and from 12.0 to 10.9 (a reduction of 1.1 mm) for first‐step pain (i.e., the CIs are narrower indicating enhanced precision in the estimates). Comparisons of the old and revised MIDs for the VAS for average pain and first‐step pain are included in Figure 3.

**FIGURE 4:**
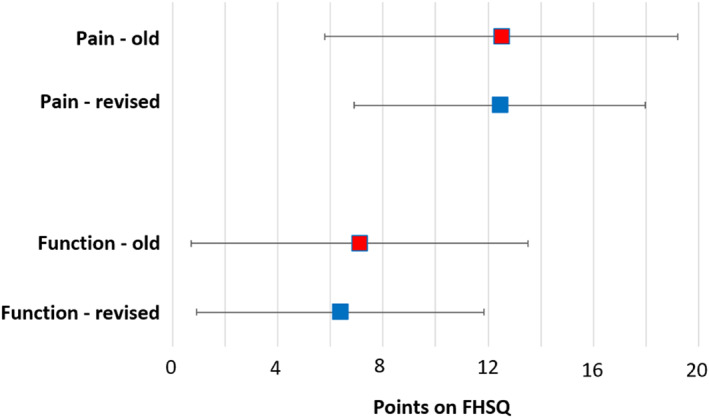
Comparisons of the old and revised MIDs for the FHSQ domains of foot pain and foot function. FHSQ, Foot Health Status Questionnaire; MID, minimal important difference.

For the FHSQ, the MID was calculated for the domains of foot pain and foot function. The MID was found to be 12.4 points for the foot pain domain (i.e. an improvement in foot pain of 12.4 points) and 6.4 points for the foot function domain (i.e. an improvement in foot function of 6.4 points). Again, the values for the foot pain and foot function domains are similar to our previous study, which determined the MID values to be 12.5 points for foot pain and 7.1 points for foot function [24]. Similar to the VAS findings presented above, the width of 95% CIs for these MIDs reduced from 13.4 to 11.1 (a reduction of 2.3 points) for foot pain and from 12.7 to 11.0 (a reduction of 1.7 points) for foot function (i.e., the CIs are narrower indicating enhanced precision in the estimates). Comparisons of old and revised MIDs for the two domains of the FHSQ that we could calculate are included in Figure 4.

It is worth noting that we chose not to calculate MIDs for the footwear and GFH domains as we did not find appreciable correlations between the 15‐point global rating of change Likert scale and the footwear or the GFH domains. This finding indicates that there is little relationship in what these FHSQ domains measure relative to the 15‐point global rating of change Likert scale when used for plantar heel pain, and as a consequence, MID calculations for these two domains would not be credible. For the footwear domain, this may simply indicate that the effect of treatment of plantar heel pain is unimportant relative to the fit of footwear. Alternatively, it may indicate that some of the interventions used in the RCTs that we pooled data from may have negatively influenced shoe fit, whereas the other interventions may have positively influenced shoe fit. For the GFH domain, it is harder to explain the lack of appreciable correlation; however, the validity of this domain has been questioned previously as it potentially suffers from a lack of discrimination or responsiveness [44]. Importantly, these two FHSQ domains—footwear and GFH—are generally not considered as important for plantar heel pain as the domains of foot pain and foot function and they have not been used in sample size calculations for previous RCTs that have evaluated interventions for plantar heel pain [30, 31, 32, 45, 46].

In relation to generalisability of our findings, the participants from the four RCTs whose data were pooled for this study can be considered generalisable to the population of individuals with plantar heel pain (i.e., on average, they are middle‐aged, predominantly female, and overweight or obese). Although our findings are generalisable to the broad plantar heel pain population, the sample size we could access does not allow us to more specifically generalise to sub‐populations of plantar heel pain because the group sizes for a ‘small change’ and ‘no change’ would be too small. Accordingly, we cannot provide precise MID estimates for individuals of different ages or BMI ranges, for example. Furthermore, as with many RCTs conducted via universities, the duration of symptoms of participants in these RCTs is relatively long (median 6–13 months), so the average individual in clinical practice may have a shorter duration of symptoms, which may affect their MIDs, but we would envisage this to be insignificant. In addition, the most recent RCT we used data from [33] used a social media recruitment strategy (Facebook), which the other earlier RCTs [30, 31, 32] did not; this led to a younger mean age in this RCT (44 years compared to 50–53 years for the earlier RCTs). We do not believe this to have negatively influenced the generalisability of our findings; indeed, it may strengthen generalisability as it is more representative of the overall population with plantar heel pain. Therefore, the MID values calculated in this study may be used to interpret results from a 100 mm VAS (for average and first‐step pain) and the FHSQ (foot pain and foot function domains) when assessing outcomes of treatment of plantar heel pain. For example, if an intervention leads to an improvement of substantially less than 12 points (e.g. an improvement of only 5 points) in the pain domain of the FHSQ, then the improvement is most likely too small for patients to detect as an important change. In the research setting (e.g. in a clinical trial), this would be regardless of whether the effect of the intervention on the outcome measure is shown to be statistically significant. This also highlights the importance when evaluating an intervention in a clinical trial of considering both clinical significance (i.e. the MID) as well as statistical significance. Importantly, a statistically significant finding in a clinical trial may not always indicate that participants have experienced an important change from their perspective.

Findings from this study may also be used to calculate sample sizes for future clinical trials that evaluate interventions for plantar heel pain. Prospective sample size calculations are fundamental to the planning of any clinical trial (e.g. RCT) and an appropriately large sample size leads to the trial having sufficient statistical power to detect important changes if they do occur. In addition, the MIDs we have calculated can be used to dichotomise participants in clinical trials in the data analysis phase to allow better interpretability of those that benefitted from treatment to those that did not (e.g. using proportions or number needed to treat), which may be substantially easier to understand compared to mean changes in health status measures [21].

This study has several strengths. The study has enhanced the validity and precision of the MID estimates (i.e., it used best‐practice methods and has provided narrower 95% CIs, which provide greater confidence in the MID values). To achieve this, we used best practice for calculating the MIDs, which included the anchor‐based approach and ensuring appreciable correlations between the anchor and outcome measures. These approaches ensure that the self‐reported experiences of participants (patients) are relied on, not distributions from statistical analyses [28, 29], and they add credibility to the MID calculations [27]. The study also used data from four high‐quality RCTs with 369 participants (up from two RCTs with 184 participants in our previous calculation [24]) and outliers were appropriately handled to ensure that data were normally distributed. Therefore, these revised MIDs are as robust as is possible with the data currently available.

However, the findings of this study need to be considered with two limitations in mind. Firstly, although we had substantially more participants in this study than in our previous study published in 2010 [24]—369 participants versus 184 participants—the MID estimates are still somewhat limited by the actual numbers of participants that responded ‘no change’ and a ‘small change’ on the 15‐point global rating of change Likert scale, which ranged from 58 to 81 participants depending on the outcome measure. Larger numbers of participants in these groups would lead to greater precision when calculating the MIDs [29]. Secondly, the results of this study are generalisable to plantar heel pain but may not be generalisable to other foot conditions (e.g. intermetatarsal neuroma) or even for plantar heel pain secondary to systemic conditions such as inflammatory arthritis (e.g. peripheral spondyloarthritis). Therefore, the MID values may not be able to be generalised to other foot conditions. Ideally, future research will investigate the MIDs for the VAS and FHSQ for other common foot conditions. In addition, the data used from the four RCTs only assessed relatively conservative treatments for plantar heel pain, so substantially more invasive or burdensome treatments such as surgery may have different MIDs.

## 5 CONCLUSION

This study re‐calculated the MIDs for the VAS (average and first‐step pain) and the FHSQ (pain and function domains). The revised MIDs have enhanced validity and precision compared to previous estimates, which will improve the interpretation of changes in the VAS and the FHSQ when used in the treatment of individuals with plantar heel pain. Clinicians can use these revised MID values to determine changes in foot health that are important to a patient after an intervention has been implemented. In addition, researchers can use these revised MID values when planning clinical trials for prospective sample size calculations and to dichotomise participants into those that benefitted from treatment and those that did not.

**Abbreviations**
CI: confidence interval
FHSQ: Foot Health Status Questionnaire
FOs: foot orthoses
GFH: general foot health
IQR: interquartile range
MID: minimal important difference
RCT: randomised controlled trial
SD: standard deviation
VAS: visual analogue scale

## AUTHOR CONTRIBUTIONS

**Karl B. Landorf:** Conceptualisation; data curation; formal analysis; investigation; methodology; project administration; supervision; validation; visualisation; writing—original draft preparation; writing—review & editing. **Georgia N. Twyford:** Data curation; formal analysis; investigation; visualisation; writing—original draft preparation; writing—review & editing. **Matthew P. Cotchett:** Formal analysis; supervision; writing—review & editing. **Glen A. Whittaker:** Formal analysis; investigation; supervision; validation; writing—review & editing.

## CONFLICT OF INTEREST STATEMENT

Karl Landorf is an Emeritus Editor and is a member of the Editorial Board of the Journal of Foot and Ankle Research. Matthew Cotchett is a member of the Editorial Board of the Journal of Foot and Ankle Research. It is the journal policy that editors are removed from the peer review and editorial decision‐making processes for manuscripts they have co‐authored. Otherwise, the authors declare that they have no conflicts of interest.

## ETHICS STATEMENT

Ethical approval was obtained from the relevant institutional ethics committees (University of Western Sydney Ethics Review Committee (Human Subjects)—approval # HREC 04/157, La Trobe University Research Ethics Committee—approval # 09–062, and La Trobe University Human Ethics Committee—approval # 15–120. All participants gave written informed consent to participate in the original RCTs.

## CONSENT FOR PUBLICATION

Not applicable.

## Supporting information

Figure S1

## Data Availability

The data analysed during this study are available from the corresponding author upon reasonable request.
