# Supplementary material for: Revised minimal important difference values for the visual analogue scale and Foot Health Status Questionnaire when used for plantar heel pain
Source: J Foot Ankle Res. 2024 Dec 16;17(4):e70021. doi: 10.1002/jfa2.70021 (PMC11649508; doi:10.1002/jfa2.70021)
Supplement: Supplementary file 1 — Figure S1 [file JFA2-17-e70021-s001.docx]

**Supporting Information S1**. Step-by-step guide for calculating minimum important difference values for patient-reported outcome measures

**Step 1**

Access appropriate longitudinal studies (e.g. RCTs) that collected data from a suitable ‘anchor’ (e.g. a 15 point GROC Likert scale^†^) and PROM of interest (e.g. FHSQ)

**Step 2**

Retrieve participants’ GROC data from short-term follow-up (e.g. ≤4 weeks after baseline)

**Step 3**

Isolate participants who reported a ‘small change’ (+2 or +3) and those who reported ‘no change’ (0 or +1) on the 15 point GROC Likert scale into two groups (group a and group b, respectively)

**Step 4**

For each participant in groups a and b, retrieve their corresponding PROM data (e.g. FHSQ – all domains) measured at baseline and the short-term follow-up

**Step 5**

For groups a and b, calculate the mean change for each domain of the PROM from baseline to the short-term follow-up (i.e. ∆ $\bar{X}$_a_ and ∆ $\bar{X}$_b_)

For groups a and b, calculate the mean change in patient-reported outcome data from baseline to the short-term follow-up (i.e. ∆ $\bar{X}$_a_ and ∆ $\bar{X}$_b_)

**Step 6**

Check normality of mean change for each domain of the PROM for groups a and b, and deal with if necessary (e.g. remove outliers) to ensure normal distribution

**Step 7**

Check correlations between the anchor (e.g. 15 point GROC Likert scale) and the mean change for each domain of the PROM for groups a and b

**Step 8**

If appreciable correlation is established^‡^, subtract the mean change for group b (‘no change’) from the mean change for group a (‘small change’) to calculate the MID (i.e. ∆ $\bar{X}$_a_ - ∆ $\bar{X}$_b_)

**Step 9**

Calculate the 95% CIs to accompany the MID (using adjusted group sample sizes if outliers are removed as per Step 6)

**Abbreviations:** CI = confidence interval, FHSQ = Foot Health Status Questionnaire, GROC = global rating of change, MID = minimum important difference, PROM = patient-reported outcome measure, RCTs = randomised controlled trials.

**Symbols:** ^†^see main article for an example of a 15 point GROC Likert scale, ∆ $\bar{X}$_a_ = mean change in PROM for group a, ∆ $\bar{X}$_b_ = mean change in PROM for group b, ^‡^appreciable correlation of at least ±0.5 needs to exist between the anchor and the PROM at follow-up, and the anchor and the change in the PROM (i.e. from initial assessment to follow-up), and ideally, these correlations should be approximately equal and opposite (as per Devji et al. 2020 *BMJ* 369: m1714) – if appreciable correlation does not exist for any domains, do not calculate MIDs for those domains.
